# P97/VCP ATPase inhibitors can rescue p97 mutation-linked motor neuron degeneration

**DOI:** 10.1093/braincomms/fcac176

**Published:** 2022-07-06

**Authors:** F Wang, S Li, T Y Wang, G A Lopez, I Antoshechkin, T F Chou

**Affiliations:** Division of Biology and Biological Engineering, California Institute of Technology, Pasadena, CA 91125, USA; Division of Biology and Biological Engineering, California Institute of Technology, Pasadena, CA 91125, USA; Proteome Exploration Laboratory, Beckman Institute, California Institute of Technology, Pasadena, CA 91125, USA; Division of Biology and Biological Engineering, California Institute of Technology, Pasadena, CA 91125, USA; Division of Biology and Biological Engineering, California Institute of Technology, Pasadena, CA 91125, USA; Division of Biology and Biological Engineering, California Institute of Technology, Pasadena, CA 91125, USA; Proteome Exploration Laboratory, Beckman Institute, California Institute of Technology, Pasadena, CA 91125, USA

**Keywords:** amyotrophic lateral sclerosis, motor neuron disease, inclusion body myopathy associated with paget disease of bone and frontotemporal dementia, p97 inhibitor, cell cycle

## Abstract

Mutations in p97/VCP cause two motor neuron diseases: inclusion body myopathy associated with Paget disease of bone and frontotemporal dementia and familial amyotrophic lateral sclerosis. How p97 mutations lead to motor neuron degeneration is, however, unknown. Here we used patient-derived induced pluripotent stem cells to generate p97 mutant motor neurons. We reduced the genetic background variation by comparing mutant motor neurons to its isogenic wild type lines. Proteomic analysis reveals that p97^R155H/+^ motor neurons upregulate several cell cycle proteins at Day 14, but this effect diminishes by Day 20. Molecular changes linked to delayed cell cycle exit are observed in p97 mutant motor neurons. We also find that two p97 inhibitors, CB-5083 and NMS-873, restore some dysregulated protein levels. In addition, two p97 inhibitors and a food and drug administration-approved cyclin-dependent kinase 4/6 inhibitor, Abemaciclib, can rescue motor neuron death. Overall, we successfully used iPSC-derived motor neurons, identified dysregulated proteome and transcriptome and showed that p97 inhibitors rescue phenotypes in this disease model.

## Teaser

Wang *et al*. investigated differential expression of RNA and protein in induced pluripotent stem cells derived motor neurons by comparing disease-causing mutant cells to its isogenic control. Two p97 inhibitors and a cyclin-dependent kinase 4/6 inhibitor restored some dysregulated protein levels and prevented motor neuron death of a disease-causing p97 mutant.

## Graphical Abstract

**Graphical abstract:**
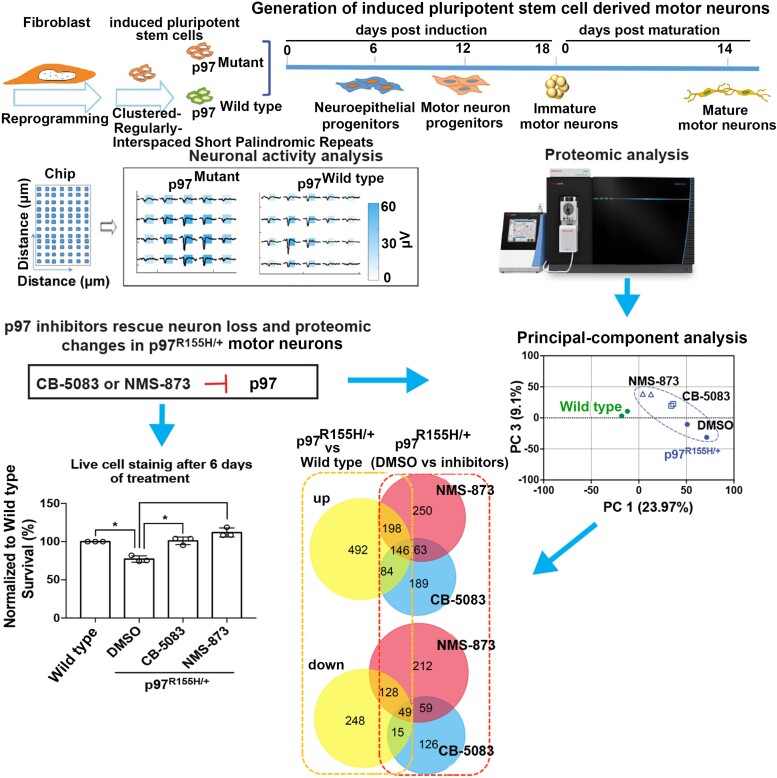

## Introduction

Human p97/VCP protein is part of the AAA+ ATPase protein family.^1^ p97 is ubiquitously expressed and is essential for a variety of cellular activities, including protein homeostasis, mitochondrial quality control, Golgi reassembly and autophagy.^1,2^ Mutations in p97 cause inclusion body myopathy associated with Paget disease of bone and frontotemporal dementia (IBMPFD), (also known as multisystem proteinopathy 1, MSP1; OMIM 167320). To date, more than 40 mutations at 29 different positions in the p97 gene have been found in IBMPFD/ALS.^3^ The most common mutation in p97 is R155H, and this change accounts for 50% of clinical cases.^4^

Symptoms of IBMPFD include adult-onset muscle weakness (myopathy), early-onset Paget disease of bone and premature frontotemporal dementia (FTD).^5^ One-third of IBMPFD patients will develop FTD.^6^ In addition, p97 is associated with 1–2% of cases of family and sporadic ALS, characterized by motor neuron (MN) degeneration.^7^ Approximately 10% of individuals with IBMPFD have a previous diagnosis of ALS.^8,9^ Both IBMPFD and ALS lead to MN degeneration.^10^ p97 mutant neurons develop vacuoles and inclusions, accumulate ubiquitinated proteins, and show aberrant localization of the DNA-binding protein, TDP-43, to the cytosol.^11–15^ A recent study found that the p97 mutation (D395G) impairs disaggregation of PHF-tau, a possible disease-linked mechanism.^16^ However, these findings either use mouse models or post-mortem tissue, which reflect some pathological features, but may fail to recapitulate the mechanisms that cause human disease.

Human-induced pluripotent stem cells (iPSCs) are a valuable tool for the study of neurological disorders.^17^ Patient-derived iPSCs have been used successfully to model a range of neurological conditions, including ALS, Alzheimer’s disease and Parkinson’s disease.^18–21^ Hall *et al.*^22^ modelled p97-related ALS using patient-derived iPSCs and revealed that cytoplasmic TDP-43, ER stress, mitochondrial function and oxidative stress are key differences between healthy control and p97 mutants MNs. While their findings suggest possible disease-linked mechanisms, the global effects of p97 mutants on human MNs remain ambiguous. Since the age of onset and clinical features vary across IBMPFD patients,^5^ genetic background effects should also be taken into account when searching for disruption of cellular functions linked to disease. Moreover, p97-related IBMPFD is thought to occur through gain-of-function. Indeed, disease-associated p97 mutants show enhanced ATPase activity and increased binding to cofactors.^23–25^ In addition, muscle phenotypes in a *Drosophila* model are rescued by p97 inhibition.^26^ Whether p97 inhibitors rescue p97 mutant-driven neurodegeneration in human MNs has yet to be evaluated.

To uncover the molecular mechanisms that underlie the effects of mutant p97 on human MNs and search for potential treatments, we differentiated iPSCs derived from IBMPFD patients. These cells, which carry the p97^R155H/+^ mutation, were compared to isogenic wild type (isoWT) control MNs. We evaluated MN survival and relevant p97 cellular functions. Proteomic and transcriptomic analyses were also performed to explore the broader effects of this disease-linked p97 mutation in human MNs. Lastly, we tested the protective effects of p97 inhibitors on p97^R155H/+^ MNs and found that p97 inhibitors rescue the neuron loss and restore dysregulated protein levels in p97^R155H/+^ MNs. Our results provide insight into the effects of p97 mutants in MNs and suggest a possible new therapeutic avenue for treating IBMPFD.

## Materials and methods

### Induced pluripotent stem cells reprogramming and maintenance

Human fibroblast from patient with p97^R155H/+^ mutant and healthy control were obtained from the Coriell Institute (Coriell code: GM21851 and GM22246) and reprogrammed into iPSCs as a published protocol using episomal vectors.^27^ Briefly, five non-integrating episomal vectors pCE-hOCT3/4, pCE-hSK, pCE-hUL, pCE-mp53DD and pCXB-EBNA1 purchased from Addgene were introduced into fibroblasts using the Human Dermal Fibroblast Nucleofector Kit (Lonza, cat# VPD-1001) and Nucleofector 2b Device (Lonza, cat# AAB-1001, programme U-023) according to the manufacturer’s protocol. The cells were then cultured on Matrigel (BD Biosciences, cat# 354230) coated 6-well plates in DMEM (containing 15% FBS and 10 ng/mL FGF) for 2 days and in E7 medium for another 8 days. Cells were cultured in mTeSR1 medium (STEMCELL, cat# 85850) from Day 10 until iPSC colonies appeared. Colonies were picked and cultured in mTesR1 with 10 nM/mL rock inhibitor (Sigma, cat# Y0503) on Matrigel-coated plates. After 24 h, the rock inhibitor was removed, and the medium was changed every day. The iPSCs were passaged using ReLeSR (STEMCELL, cat# 85872) every 5 to 7 days at split ratios of 1:6 to 1:9 when they reached ∼80% confluence. Cells were tested for mycoplasma routinely using MycoAlert Mycoplasma Detection Kit (Lonza, cat# LT07-118). Quantitative RT-PCR (qPCR) method followed manufacturer instructions for the hPSC Genetic Analysis Kit (Stemcell, cat# 07550) to detect recurrent karyotypic abnormalities reported in iPSCs. Clones that passed the RT-PCR assay were sent for karyotyping (ThermoFisher, KaryoStat™ assay) after 10 passages.

### CRISPR/cas9-mediated genome editing of induced pluripotent stem cells

Guide RNA, Alt-R® CRISPR-Cas9 tracrRNA ATTO™ 550 (IDT, cat# 1075928) and Alt-R® S.p. HiFi Cas9 Nuclease V3 (IDT, cat# 1081061) were used to form ribonucleoprotein (RNP) complexes following the manufacturer’s protocol. The H155R single-strand donor DNA sequence (H155R-Reverse Complement) contains a T to C correction at the R155H mutation site of the human p97 gene. The donor sequence includes a silent G to T mutation at the PAM site to avoid re-cutting by Cas9. Another silent mutation, C to G, brings in the SphI digestion site to identify edited clones. Similarly, a R155H single-strand donor DNA sequence (Supplementary Table S1) was used to convert WT cells into isop97^R155H/+^ cells. The iPSC medium was changed for IPSCs (50–80% confluence) with fresh mTeSR1 containing 10 μM rock inhibitor and 5 μM L755507 (Sigma, cat# SML1362) 1 day before electroporation. RNP complexes and the single-strand donor DNA were transfected into the iPSCs using the Human Stem Cell Nucleofector Starter Kit (Lonza, cat# VPH-5002) and Nucleofector 2b Device (Lonza, cat# AAB-1001, programme B-016) according to manufacturer instructions. Cells were plated on Matrigel-coated 6-well plates in mTesR1 medium containing clone R supplement (STEMCELL, cat# 05888) at low density. After 24 h, the cells were observed with a fluorescence microscope to confirm the RNP protein was transfected into the cells. Clone R was removed after 48 h. Colonies were picked 7–10 days after transfection and cultured on Matrigel-coated 24-well plate in mTesR1 medium containing 10 μM rock inhibitor for another 7–10 days. Then individual colonies were manually split into two halves. One half was used for genomic DNA extraction with QuickExtract™ DNA Extraction Solution (Epibio, cat# BQ0901S). The other half was maintained in 24-well plates. PCR reactions were performed to amplify regions covering the R155H mutation site using VCP-Ex5 (F + R) primers and Platinum Taq DNA polymerase (Invitrogen, cat# 10966018) following the user guide. The PCR products were digested using the SphI restriction enzyme (NEB, cat# R3182). Clones that could be digested by SphI were further sequenced by Laragen DNA Sequencing Service. Sequences of the guide RNA, single-strand donor DNA sequences, and PCR primers are listed in Supplementary Table S1.

### Motor neuron differentiation and maturation

We followed a previously published protocol^28^ to differentiate human iPSCs into MNs. We first prepared basal induction medium which contained advanced DMEM/F12 (Gibco, cat# 12634-010) and neurobasal medium (Gibco, cat# 21103-049) (1:1 v/v), 1% 50× B27 (Gibco, cat# 17504-044), 0.5% 100× N2 (Gibco, cat# 17502-048), 0.1 mM Ascorbic Acid (Sigma, cat# A4544), 1% 100× Glutamax (Gibco, cat# 35050-061) and 1% 100× Antibiotic-Antimycotic (Gibco, cat# 15240-062). When iPSCs reached ∼80% confluence, they were in basal induction medium containing 3 μM CHIR99021 (Cayman Chemical, cat# 13122), 2 μM SB431542 (Cayman Chemical, cat# 13031) and 2 μM DMH-1 (Tocris, cat# 4126), plated on Geltrex (Gibco, cat# A1413201) coated 6-well plates and cultured for 6 days to generate NSC cells. We changed the culture medium every other day. The NSC cells were then dissociated with Accutase (STEMCELL, cat# 07920) and further induced by culturing with the induction basal medium containing 1 μM CHIR99021, 2 μM SB431542, 2 μM DMH-1, 0.1 μM RA (Sigma, cat# 554720), and 0.5 μM purmorphamine (R&D, cat# 4551) to become OLIG2^+^ motor neuron progenitors (MNPs). The medium was changed every other day. MNPs can be expanded for several passages with the induction basal medium containing 3 μM CHIR99021, 2 μM DMH-1, 2 μM SB431542, 0.1 μM RA, 0.5 μM Purmorphamine, and 0.5 mM VPA (R&D, cat# 2815), and split 1:6 every 6 days with Accutase. MNPs were dissociated with Accutase, transferred into poly-Hema (Sigma, cat# P3932) coated flasks, and treated with basal induction medium containing 0.5 μM RA and 0.1 μM purmorphamine for another 6 days on the shaker to let the cells form HB9^+^ EBs (MN-sus) and expand. We changed half of the medium every other day. MN-sus can be dissociated into single cells with Accutase and froze for future maturation culture.

The maturation of MNs was followed using a 14-day protocol from BrainXell. Briefly, the MNs were thawed and plated on poly-lysine (Sigma, cat# P7886) coated plates in MN maturation medium containing DMEM/F12 (Gibco, cat# 11330-032) and neurobasal medium (1:1 v/v), 2% 50× B27, 1% 100× N2, 0.25% 100× Glutamax, 10 ng/mL BDNF (Peprotech, cat# 450-02), 10 ng/mL GDNF (Peprotech, cat# 450-10), 1 ng/mL TGF-β1 (Peprotech, cat# 100-21c), and 1× Brainfast supplement (BrainXell). The medium was changed with the same medium containing 15 μg/mL geltrex on Day 1. An equal volume of maturation medium (excluding Geltrex) was added on Day 4. Half of the medium (excluding the Brainfast supplement and Geltrex) was changed twice weekly from Day 7. The MNs can be maintained for at least 3 weeks. For the p97 inhibitor treatment assays, we changed medium with fresh medium containing 400 nM of CB-5083 or NMS-873 (purchased from MedKoo; CB-5083, cat# 206489; NMS-873, cat# 406458), or same volume of DMSO at D14, then incubate for 6 days and harvested cells on D20.

### Quantitative real-time PCR

Cells were harvested and pellest resuspended in DPBS/TRIzol-LS mixture (Ambion, cat# 10296010; v/v 1:3). Total RNA samples were extracted from the TRIzol-LS mixture using Direct-zol RNA MiniPrep plus kit (Zymo Research, cat# R2072) according to the manufacturer’s instructions. The RNA concentration was measured with NanoDrop Lite UV visible spectrophotometer (Thermo Scientific, cat# S/N 2361). One microgram of the total RNA was used to reverse transcribe complementary DNA using the SensiFAST™ cDNA Synthesis Kit (Bioline, cat# BIO-65054). qRT-PCR reactions were performed using SensiFAST Probe HI-ROX Mix (Bioline, cat# BIO-82020) on the QuantStudio™ 5 Real-Time PCR System (Thermo Scientific, cat# A28140). 2^(-delta CT) was calculated by normalizing GAPDH levels. All sample reactions were carried out in triplicate. The error bar reveals the standard deviation of the mean from all of the cell lines used in this paper. The primer probes used in this study are provided in Supplementary Table S2.

### Immunocytochemistry staining and imaging

The iPSCs were stained using an Alkaline Phosphatase Staining Kit (StemTAG, cat# CBA-300) and PSC Immunocytochemistry Kit (Invitrogen, cat# A24881) by following the manufacturer’s protocol. NSC cells were stained using the Human Neural Stem Cell Immunocytochemistry Kit (Invitrogen, cat# A24354) following the manufacturer’s protocol. MNP and MN cells were fixed with 4% PFA at room temperature for 15 min or with cold methanol for 5 min, blocked with 10% donkey serum and 0.1% Triton X-100 in 1× DPBS for 1 h. The cells were followed incubated with the primary antibodies (Supplementary Table S3) for 2 h at r.t., washed three times with DPBS containing 0.1% BSA, and incubated with species-specific Alexa Fluor 488-conjugated secondary antibody (donkey anti-mouse immunoglobulin G (IgG), 1:500, Life Technologies) or Alexa Fluor 555-conjugated secondary antibody (Rabbit anti-goat IgG, 1:500, Life Technologies) for 30 min. Cells were then washed three times with DPBS and nuclei stained using Hochest (10 ng/mL) for 10 min. Cell images were acquired using the EVOS FL Auto 2 Imaging System (Invitrogen, cat# AMAFD2000).

### Western blot

MN cells were scraped off plates, washed with DPBS, and centrifuged at 300 g for 4 min to remove supernatant. Pellets were frozen at −80°C. Pellets were resuspended with 100 μL lysis buffer (50 mM Tris-HCl pH 8.0, 150 mM NaCl, 1% Triton X-100 with protease inhibitor tablet, 50 μM MG132 and 50 mM NEM), and incubated on ice for 10 min with occasional vortexing. After that, samples were centrifuged at 15 000 rpm at 4°C for 10 min and transferred 90 μL cell lysate into a 1.5 mL tube. Total soluble protein concentrations were measured using the Bradford test (Bio-Rad, cat# 5000006). Next we added 30 μL 4× Laemmli sample buffer (Bio-Rad, cat# 161-0774) was added, and samples were boiled for 5 min. An equal amount of protein was loaded and separated using 4–20% Mini-PROTEAN TGX precast gels (Bio-Rad, cat# 456-1096) and transferred to nitrocellulose membranes using Trans-Blot Turbo Transfer System (Bio-Rad, cat# 170-4155). Membranes were blocked with 1× TBST with 5% w/v non-fat milk, incubated with primary antibodies for 2 h at r.t. or overnight at 4°C and incubated with HRP-conjugated secondary antibodies (Bio-Rad, 1:3000 dilution) for 2 h at room temperature. Then ECL reagent (MilliporeSigma, cat# WBKLS0500) and ChemiDoc MP Imaging System (Bio-Rad) were used to image the blots. The blot densities were analyzed using Image Lab 6.0.1 software (Bio-Rad). Primary antibodies used in this study are listed in Supplementary Table S1, and uncropped western blot images are shown in Supplementary Fig. S6.

### Motor neuron cell survival assay

To compare p97 WT and R155H/+ cells, MNs were thawed and plated on poly-lysine coated 96-well plates (Greiner, cat# 655090) at a density of 1000 cells per 100 μL per well in MN maturation medium as described in the ‘MN differentiation and maturation’ section. We changed medium with 100 μL of the same medium containing 15 μg/mL geltrex on Day 1. An additional medium of 100 μL (excluding Geltrex) was added on Day 4. Half of the medium (excluding the Brainfast supplement and Geltrex) was changed on Day 7 and 11. Ara-C of 200 nM was added to inhibit non-MN cells from proliferating. On Day 14, we replaced the medium with N2 medium (DMEM/F12 and neurobasal medium, 1:1 v/v, 1% 100× N2) and changed the N2 medium every 6 days. Cell viability was monitored every 3 or 4 days by staining with Calcein AM Viability Dye (Thermo, cat# 65-0853-81), acquiring and analyzing images with ImageXpress Micro Confocal High-Content Imaging System (Molecular Devices).

For p97 inhibitor treatment, MNs were plated on poly-lysine coated 384-well plates (Greiner, cat# 781946) at a density of 500 cells per 30 μL per well. Use the same medium for plating and maintaining cells. We monitored cell viability at Day 1 and Day 14. On Day 14, we replaced the medium with N2 medium containing DMSO or p97 inhibitors (CB-5083 and NMS-873) or Abemaciclib and tested cell viability after 6 days of treatment.

### Electrophysiological recordings

We pre-coated the recording electrode area containing 26 400 platinum microelectrodes on the MaxTwo HD-MEA 6-well plate (MaxWell Biosystems AG) with poly-lysine. MN cells of 2 × 10^5^ and 4 × 10^4^ astrocytes (purchased from iCell) were plated per well on the poly-lysine coated area. Medium and culture methods are the same as those described in the ‘motor neuron differentiation and maturation’ section. Spontaneous electrical activity and networks were analyzed using the MaxTwo microelectrode array system (MaxWell Biosystems AG).

### Proteomics

After culture, MN cells were scraped off the plates, washed with DPBS and centrifuged at 300 g for 4 min to remove the supernatant. Pellets were frozen at −80°C. The mass spec samples were prepared by following instructions for the EasyPep Mini MS Sample Prep Kit (Thermo Scientific, cat# A4006). Samples were dried using a vacuum centrifuge and resuspended in 0.1% formic acid (Thermo, cat# 85178) water solution. Peptide concentrations were measured using the Quantitative Fluorometric Peptide Assay kit (Thermo, cat# 23290).

LC-MS/MS experiments were performed by loading 0.5 μg sample onto an EASY-nLC 1000 (Thermo Scientific) connected to an Orbitrap Eclipse Tribrid mass spectrometer (Thermo Scientific). Peptides were separated on an Aurora UHPLC Column (25 cm × 75 µm, 1.6 µm C18, AUR2-25075C18A, Ion Opticks) with a flow rate of 0.4 μL/min and a total duration time of 131 min following the gradient composed of 3% Solvent B for 1 min, 3–19% B for 72 min, 19–29% B for 28 min, 29–41% B for 20 min, 41–95% B for 3 min and 95–98% B for 7 min. Solvent A consists of 97.8% H_2_O, 2% ACN and 0.2% formic acid; solvent B consists of 19.8% H_2_O, 80% ACN and 0.2% formic acid. MS1 scans were acquired with a range of 400–1600 m/z in the Orbitrap at 120 k resolution. The maximum injection time was 50 ms, and the AGC target was 2 × 10^5^. The filter dynamic exclusion was set to exclude after one time, 30 s duration, and 10 ppm mass tolerance. MS2 scans were acquired with quadrupole isolation mode and higher-energy collisional dissociation activation type in the Iontrap. The isolation window was 1.4 m/z, collision energy was 35%, maximum injection time was 35 ms, and the AGC target was 1 × 10^4^. Other global settings were as follows: ion source type, NSI; spray voltage, positive ion 2400 V, negative ion 600 V; ion transfer tube temperature, 300°C. Method modifications and data collection were performed using Xcalibur software (Thermo Scientific).

Proteomic analysis was performed using Proteome Discoverer 2.4 (Thermo Scientific) software with the Uniprot human database and the SequestHT with Percolator validation. Protein abundance normalization was performed on total peptide. The data exported from PD2.4 were then used for further analysis. Limma analysis was performed using R studio following the user guide.^29^ The volcano figures were plotted using Origin 2019b. PCA analyses were generated with PD2.4 and plotted using Prism 7. The Venn diagram was performed using FunRich 3.1. The heatmap figure was performed with prism 7. Functional enrichment analyses were performed with g:Profiler.^30^ The bubble plot was generated using Origin 2019b.

### Transcriptomics

RNA samples were prepared by following the same procedures described in the ‘Quantitative real-time PCR’ method part. The integrity of RNA was assessed using RNA 6000 Pico Kit for Bioanalyzer (Agilent Technologies, cat# 5067-1513), and mRNA was isolated using NEBNext Poly (A) mRNA Magnetic Isolation Module (NEB, cat# E7490). The Ultra II RNA Library Prep Kit for Illumina (NEB, cat# E7770) was used to construct RNA-seq libraries by following the manufacturer’s instructions. Briefly,^31^ mRNA isolated from ∼1 μg of total RNA was fragmented to the average size of 200 nt by incubating at 94°C for 15 min in the first strand buffer, cDNA was synthesized using random primers and ProtoScript II Reverse Transcriptase followed by the second-strand synthesis using NEB Second Strand Synthesis Enzyme Mix. The resulting DNA fragments were end-repaired, dA tailed and ligated to NEBNext hairpin adaptors (NEB, cat# E7335). After ligation, adaptors were converted to the ‘Y’ shape by treating with USER enzyme and DNA fragments were size selected using Agencourt AMPure XP beads (Beckman Coulter, cat# A63880) to generate fragment sizes between 250 and 350 bp. Adaptor-ligated DNA was PCR amplified, followed by AMPure XP bead clean up. Libraries were quantified with Qubit dsDNA HS kit (Thermo Scientific, cat# Q32854), and the size distribution was confirmed with the High Sensitivity DNA Kit for Bioanalyzer (Agilent Technologies, cat# 5067-4626). Libraries were sequenced on Illumina HiSeq2500 in single read mode with a read length of 100 nt to the depth of 30 million reads per sample following manufacturer’s instructions. Base calls were performed with RTA 1.13.48.0 followed by conversion to FASTQ with bcl2fastq 1.8.4. Further data analyses are described in the ‘Proteomics’ section.

### Statistical analysis

Statistical analyses were carried out by one-tailed Student’s *t*-test or one-way ANOVA using Prism 7.0. Three independent biological replicates were used. *P*-values < 0.05 are reported as statistically significant and are depicted as follows throughout the manuscript: ^∗^  *P* < 0.05, ^∗∗^  *P* < 0.01, ^∗∗∗∗^  *P* < 0.0001.

### Data availability statement

All relevant data generated during this study are included in the article and in the Supplementary Information. The mass spectrometry raw data are deposited to the ProteomeXchance Consortium (https://www.ebi.ac.uk/pride/) via the PRIDE repository with the data set identifier PXD026685 and 10.6019/PXD026685’ (review login ID reviewer_pxd06685@ebi.ac.uk and password LWzWNb1o). The RNA sequencing data is available at NCBI SRA under BioProject ID (PRJNA739755). Additional raw data generated during the present study and relevant information are available from the corresponding authors upon request.

## Results

### Generation of induced pluripotent stem cells carrying R155H p97 mutation and isogenic wild type p97

**Figure 1:**
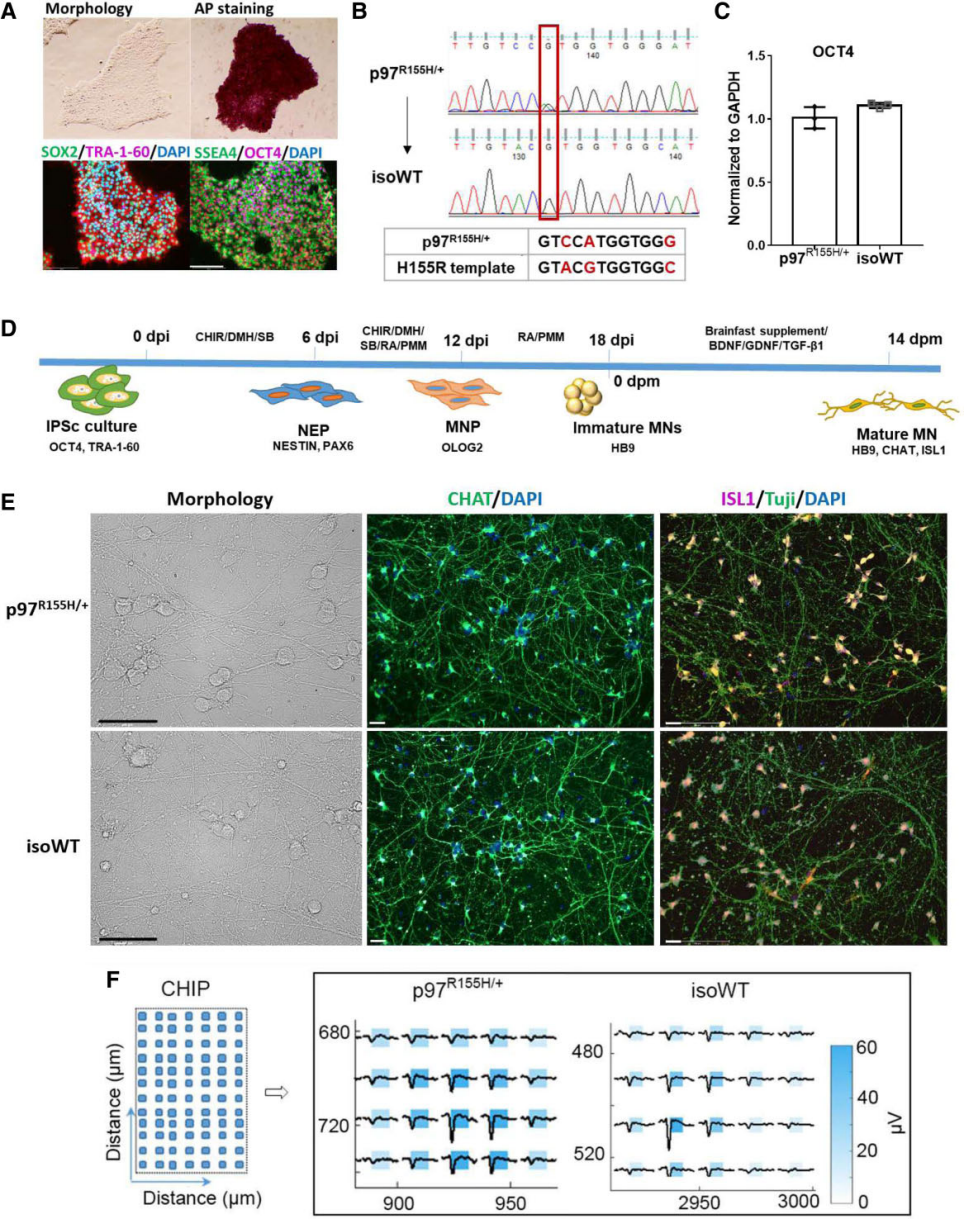
**Generation of ihMNs from patient-derived fibroblast cells.** (**A**) Verification of iPSC identity in cells derived from p97^R155H/+^ patient cells. Scale bar indicates 200 μm. (**B**) Generation of isogenic lines using CRISPR/Cas9 to correct p97 R155H mutation. Two synonymous mutations were introduced in the HDR template: the C to A mutation is to remove PAM site to prevent re-cutting by Cas9 and the G to C mutation is to introduce a restriction enzyme site (ShpI) to allow us to identify presence of HDR by a restriction fragment length polymorphism (RFLP) assay. The positive iPS clones detected by RFLP assay were further confirmed by DNA sequencing. (**C**) qPCR analysis of the OCT4 mRNA expression in p97^R155H/+^ and isoWT iPSCs. (**D**) Schematic showing strategy for promoting motor neuron differentiation. Days post induction (dpi) and Days post maturation (dpm). (**E**) Representative images of morphology and CHAT, ISL1 immunofluorescence staining in mature MNs. Scale bar indicates 50 μm. (**F**) Illustration of the chip used to recorded neuron activity in Maxwell (left, CHIP) and the representative footprints of spontaneous firing from a single cluster of MNs at 14 dpm. Immature MNs were seeded on chips in the Maxwell plate and incubated under maturation culture conditions. Neuron activity was recorded at 7, 10 and 14 dpm. Each square represents an electrode used to record neuron activity. The distance indicates the position of the neuron activity was recorded in the chip.

Neurons induced from reprogrammed patient cells are a powerful tool to explore mechanisms linked to CNS diseases, as well as to test potential therapies.^32^ To study the pathological effects of p97 mutation in human MNs, we generated human iPSCs from IBMPFD patient fibroblasts harbouring a heterozygous p97 mutation (p97^R155H/+^) through reprogramming.^33^ Cell morphology, alkaline phosphatase (AP) staining and immunofluorescence (IF) staining of four pluripotency markers (Fig. 1A) were used to characterize the iPSCs.^34^ It has been widely reported that genetic abnormalities can occur during iPSC generation and routine culture.^35^ To rule out abnormal iPSC clones, we tested for recurrent karyotypic abnormalities reported in iPSCs using qPCR^36^ (Supplementary Fig. S1A). Clones that passed this qPCR assay were sent for karyotyping after 10 passages, and the normal karyotype clones were used for differentiation (Supplementary Fig. S1B). Comparing patient-derived cells with cells from genetically different healthy controls has been reported to overlook subtle phenotypes.^37^ To eliminate the effects of genetic background, we generated isogenic iPSCs (isoWT) by correcting the p97 mutation (R155H) in patient iPSCs using CRISPR and we also converted a WT to isogenic R155H.^37^ Isogenic iPSCs were confirmed using DNA sequencing (Fig. 1B and Supplementary Fig. S3A). OCT4 RNA levels were the same in p97^R155H/+^ and isoWT iPSCs, indicating that the stem cell pluripotency of isoWT iPSCs was not affected by CRISPR (Fig. 1C).

### Differentiation of induced pluripotent stem cells into motor neurons

One patient’s iPSC line and its isogenic WT line were differentiated to MNs in triplicates using a published 18-day protocol^28^ (Fig. 1D). To capture the variability of differentiation process, we performed the experiments three times independently by two different research studies. The expression of specific markers for each differentiation stage was detected using qPCR and IF staining. Following differentiation, the expression of OCT4, which maintains and supports induction of stem cell pluripotency,^38^ was significantly decreased. The expression of the neuroectodermal stem cell marker,^39^ Nestin (NES), continually increased as cells progressed from neuroepithelial progenitors (NEPs) to MNPs. The highest expression of OLIG2 was observed in MNPs, and HB9 expression was observed in immature MNs at 18 days post induction (dpi) (Supplementary Fig. S2A). Consistent with our qPCR results, immunofluorescence signals indicated the presence of specific cell types: NES, SOX1 in NEPs, Oligo 2 in NMPs and HB9 in immature MNs (Supplementary Fig. S2B). To generate mature MNs, the immature MNs were re-plated and cultured for another 14 days using maturation media (Fig. 1D). At 14 days post maturation (dpm), we observed uniform neuron-like morphology in both p97^R155H/+^ and isoWT MNs (Fig. 1E). IF staining showed that both p97^R155H/+^ and isoWT cultures yielded > 90% CHAT and ISL1 expressing MNs (Fig. 1E). In addition, spontaneous firing was observed from both p97^R155H/+^ and isoWT MNs using the Maxwell activity scan assay (Fig. 1F and Supplementary Fig. S2C).

### p97^R155H/+^ motor neurons recapitulate neurodegeneration

**Figure 2:**
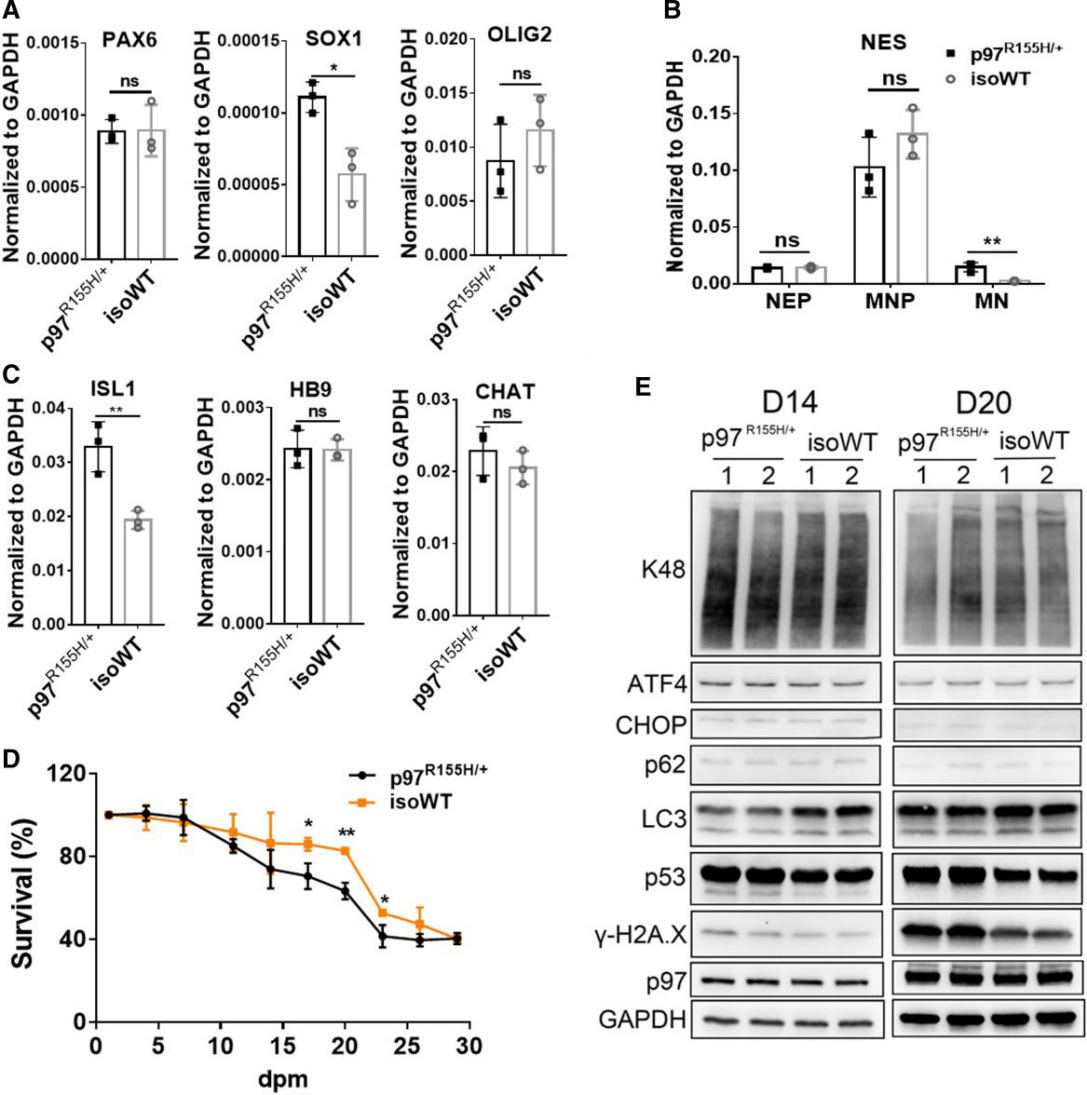
**Comparison of MN differentiation of p97^R155H/+^ and isoWT cells.** (**A-C**) qPCR analysis of specific markers at different stages of MN induction. PAX6 and SOX1 were detected at 6 dpi (NEP stage). Olig2 was detected at 12 dpi (MNP stage). ISL1, HB9 and CHAT were detected at 14 dpm (mature MNs). NES was detected at 6, 12and 14 dpm. (**D**) Cell survival curves during MN maturation. Data represent mean ± SD, *n* = 3. *: *P* < 0.05, **: *P* < 0.01 according to unpaired *t*-test. (**E**) Western blot of D14 and D20 MNs. FC indicates the average fold change (ratio of p97^R155H/+^ to isoWT). Each data point represents an independent differentiation of the same iPSC line.

To assess the influence of p97^R155H/+^ on MN differentiation, we compared the expression of *PAX6* and *SOX1* in NEPs, *Olig2* in NMPs, *ISL1, CHAT, HB9* in mature MNs and NES at all the three stages between p97^R155H/+^ and isoWT. During differentiation, the increased expression of *SOX1* was observed in p97^R155H/+^ NEPs (Figs. 2A and B). Expression of the MN-specific marker, *HB9*, and mature MN marker, *CHAT,*^28^ showed no difference between p97^R155H/+^ and isoWT MNs. However, the expression of NES (a stem cell marker) and *ISL1* (the earliest marker of developing cholinergic neurons)^40–42^ were higher in p97^R155H/+^ MNs than in isoWT MNs at 14 dpm (Figs. 2B and C).

Neuron loss and death is a pathological feature of neurodegeneration.^43^ To check whether the induced p97^R155H/+^ MNs recapitulate this neurodegeneration, a cell survival assay was performed during MN maturation culture using Calcein AM live cell staining (Fig. 2D and Supplementary Fig. S3B). p97^R155H/+^ and isoWT MNs displayed no significant difference in cell survival from 1 to 14 dpm. However, between 17 and 23 dpm, significantly decreased neuron survival was observed in p97^R155H/+^ cultures (Fig. 2D), and similar results were observed for the WT and iso p97^R155H/+^ (Supplementary Fig. S3B).

To investigate the cellular effects of p97^R155H/+^, we harvested the MNs at 14 and 20 dpm, hereafter referred to as D14 and D20 MNs. We examined markers related to proteasomal degradation, autophagy and cell death. The levels of p97, p62, proteasomal substrates (K48 poly-ubiquitinated substrates) and unfolded protein response (UPR) proteins (ATF4 and CHOP) were not affected by p97^R155H/+^ at both 14 and 20 dpm. Relative to controls, p97^R155H/+^ MNs showed decreased total LC3 levels and increases in p53 and γ-H2Ax from 14 to 20 dpm. However, the ratio of LC3-II:LC3-I in p97^R155H/+^ MNs did not change significantly at D14 but decreased slightly at D20 (Supplementary Fig. S4A). TDP43 staining displayed no difference between p97^R155H/+^ and isoWT MNs at 20 dpm (Supplementary Figs. S4B and C). Taken together, we find that two markers (p53 and γ-H2Ax) of neurodegeneration are present in mature MNs at both D14 and D20.

### Proteomic and transcriptomic profiling of mature motor neurons

**Figure 3:**
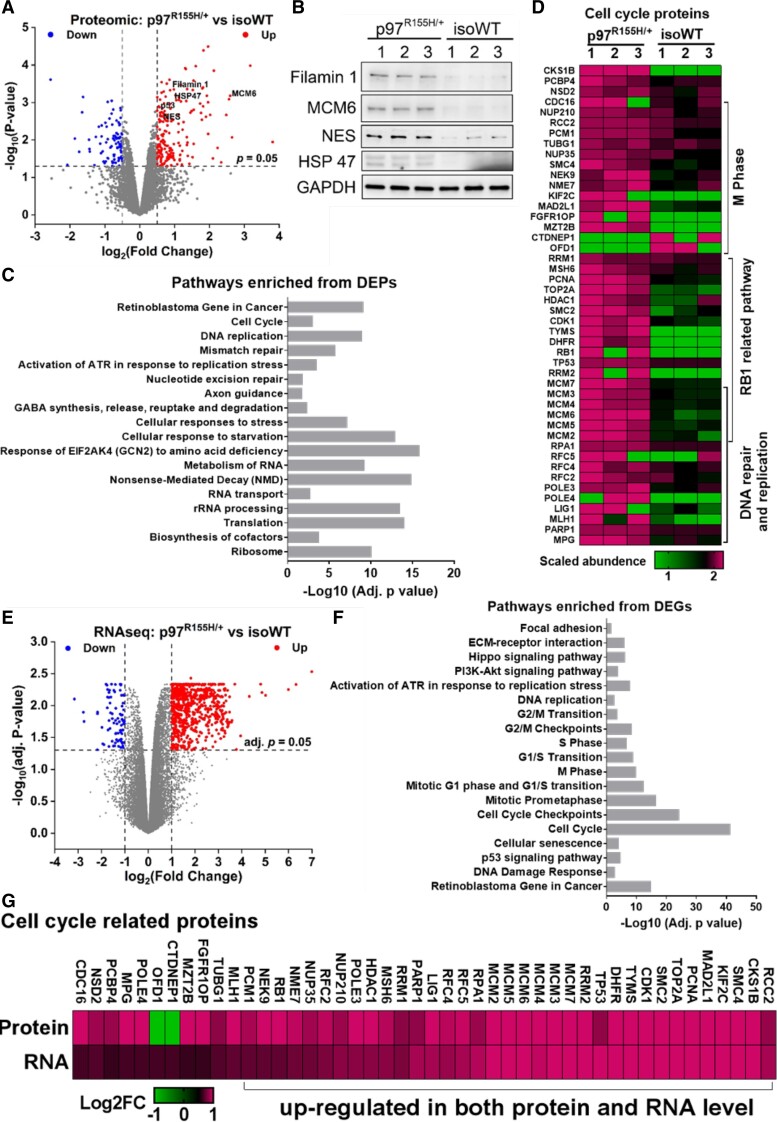
**Proteomic and transcriptomic analysis on D14 MNs.** (**A**) Volcano plot displaying the proteomic changes in p97^R155H/+^ MNs, log_2_ (Fold Change) indicates the logarithm to the base 2 of fold change, *n* = 3. (**B**) Western blot showing increases in NES, MCM6, Filamin 1 and HSP47 are consistent with proteomic data. FC indicates the average fold change in (p97^R155H/+^/isoWT). (**C**) Functional enrichment analysis on the proteins affected by p97^R155H/+^. (**D**) Heatmap displaying the scaled abundance of DEPs related to cell cycle. (**E**) Volcano plot displaying the transcriptomic changes in p97^R155H/+^ MNs. (**F**) Functional enrichment analysis on the DEGs identified from p97^R155H/+^ MNs. (**G**) Heatmap representation of a comparison between RNA-seq and proteomics for cell cycle-related DEPs. Log2FC represents log_2_ (Fold Change).

To elucidate the molecular mechanisms linked to p97^R155H/+^ driven neurodegeneration, we conducted a proteomic and transcriptomic analysis of D14 MNs to capture dysregulated markers at early time points in maturation. Three independent biological replicates were performed for both p97^R155H/+^ and isoWT MNs. The proteomic analysis was performed using label-free quantification. A total of 7101 proteins were identified (67131 peptides, FDR < 1%) and quantified from all six samples (Supplementary Table S4). After excluding data with more than 1 missing value in both groups, we performed a differential expression analysis on the remaining 6043 proteins using Limma^44^ (Supplementary Table S5). We identified 778 differentially expressed proteins (DEPs, *P* < 0.05) (Fig. 3A). Of them, ISL1, NES and p53 were increased in p97^R155H/+^ MNs. These data are consistent with our qPCR and western blot results mentioned previously (Figs. 2B–E). To further validate our proteomic data, we determined the protein levels of NES, Filamin 1, MCM6 and HSP47 by western blotting (Fig. 3B). Consistent with our proteomic data, all four proteins were increased in p97^R155H/+^ MNs. Functional enrichment analysis revealed 93 and 103 of the 778 DEPs are synapse and mitochondrion components respectively (Supplementary Table S6), which indicates that synapse and mitochondrion are potentially disrupted in p97 mutant MNs, as described previously.^22^ Cellular pathways related to translation, RNA metabolism, cellular response to stress, DNA repair and replication and cell cycle were also altered in p97^R155H/+^ MNs (Fig. 3C). In addition, many DEPs involved in DNA repair and replication and retinoblastoma protein (RB1)-related pathways overlapped with cell cycle-related DEPs. The majority of those DEPs were increased in p97^R155H/+^ MNs (Fig. 3D).

Using RNA-seq, we identified 706 differentially expressed genes (DEGs) [Adj. *P* < 0.05 and |log_2_ (fold change) >1] from 20 629 genes (Supplementary Table S7). Of the 706 DEGs, 649 were upregulated and 57 were downregulated in p97^R155H/+^ MNs (Fig. 3E). Consistent with our proteomic analysis, functional enrichment analysis on DEGs revealed that cell cycle, DNA replication and RB1-related pathways were potentially dysregulated in p97^R155H/+^ MNs (Fig. 3F, Supplementary Table S8). Of the DEGs, all 113 cell cycle-related genes were upregulated in p97^R155H/+^ MNs (Supplementary Table S9). Compared to the proteomics analysis, 35 of the 46 cell cycle-related DEPs, were upregulated on both the protein and RNA level (Fig. 3G).

### p97^R155H/+^ upregulates the RB1/E2F1 pathway in mature MNs

**Figure 4:**
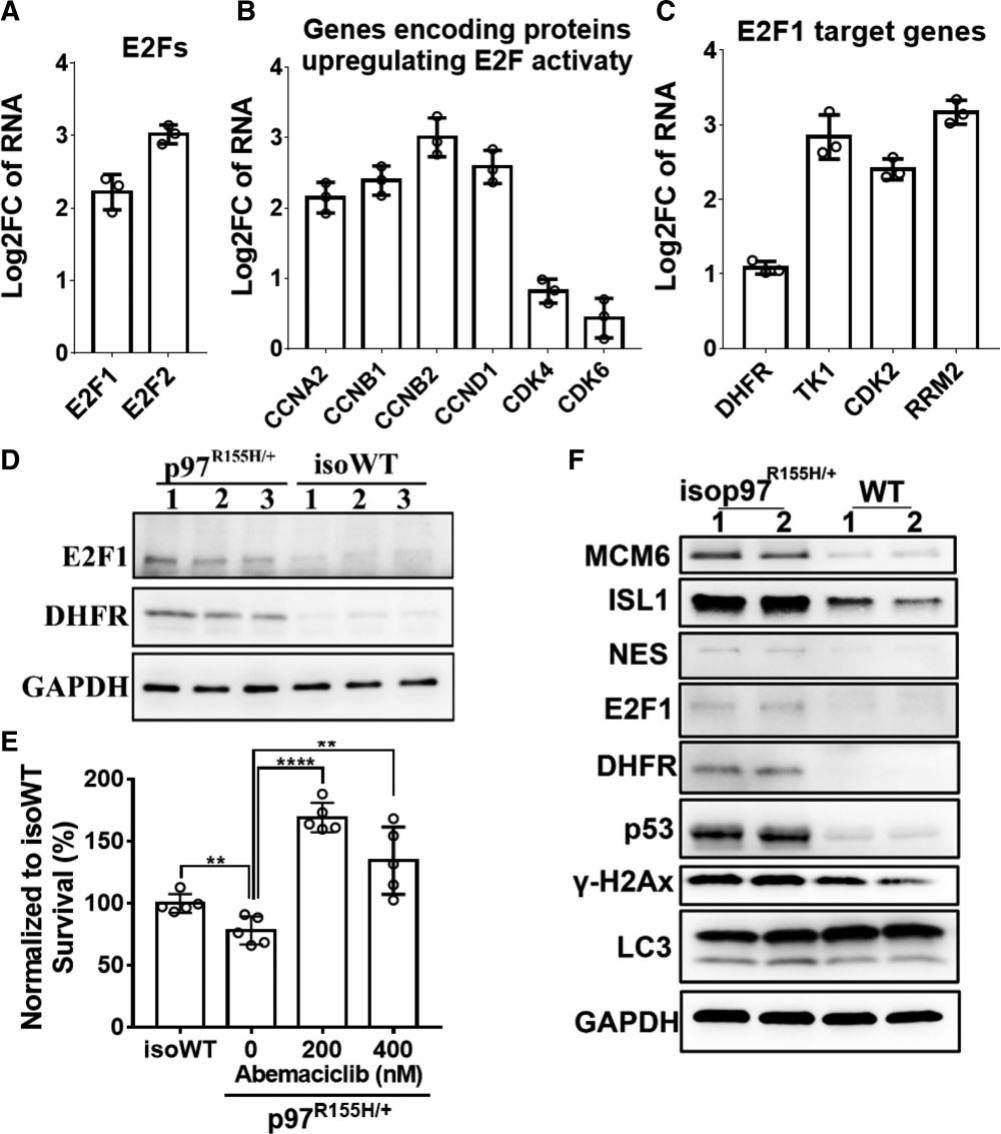
**p97^R155H/+^ activates the RB1/E2F1 pathway in mature MNs.** (**A-C**) RNA-seq data reveal upregulation of genes involved in E2F1 pathway. Log2FC represents log_2_ (Fold Change). Each data point represents an independent differentiation of the same iPSC line, *n* = 3. (**D**) Western blot showing the upregulation of E2F1 pathway in p97^R155H/+^ MNs. FC indicates the average fold change of (p97^R155H/+^/isoWT). (**E**) Cell survival results of MNs were treated with DMSO, 200 nM or 400 nM Abemaciclib. Live cell staining was performed at 26 dpm, total cell numbers were counted and normalized to isoWT MNs, *n* = 5, **: *P* < 0.01, **** *P*< 0.0001 according to unpaired *t*-test. (**F**) Western blot of D14 MNs derived from an unaffected individual carrying WT p97 (WT) and its isogeneic MNs carrying p97^R155H/+^ mutation (isop97 ^R155H/+^).

Both our proteomic and RNA-seq data revealed increased expression of cell cycle-related genes and proteins in p97^R155H/+^ MNs. This indicates that the cell cycle is potentially deregulated in mature p97^R155H/+^ MNs. The RB1/E2F pathways play important roles in cell cycle control as genes encoding DNA replication and cell cycle regulatory factors are regulated by E2Fs.^45^ The overexpression of E2F1 in quiescent cells leads to the induction of cellular DNA synthesis and apoptosis.^46,47^ Our RNA-seq data indicates that *E2F1* and *E2F2* are upregulated in p97^R155H/+^ MNs (Fig. 4A). Genes encoding proteins which positively regulate E2F pathways, including CCND1 and CDK4/6, were also upregulated in p97^R155H/+^ MNs (Fig. 4B). The upregulation of E2F1, CDKs and cyclins suggests that E2F1 transcriptional activity is increased. Indeed known E2F1 target genes, including *DHFR, CDK2, RRM2 and TK1,*^48–50^ are upregulated (Fig. 4C). In addition, CDKs and cyclins inactivate RB1 by phosphorylation and release E2F1 to induce the transcription of cell cycle genes.^51^ Moreover, the protein levels of E2F1 and *DHFR* were significant elevated in p97^R155H/+^ MNs (Fig. 4D). These data suggest that the RB1/E2F1 pathway is upregulated in p97^R155H/+^ MNs.

Cell cycle deregulation has been observed in multiple NDs, including AD, ALS and SMA.^52,53^ RB1/E2F1 pathway is also linked to cell fate decisions and the induction of apoptosis.^51^ To evaluate whether the upregulation of E2F1 pathway is associated with the cell death of p97^R155H/+^ MNs, In particular, we inhibited RB1/E2F1pathway through treatments with a FDA-approved CDK4/6 inhibitor, Abemaciclib.^54,55^ p97^R155H/+^ and iso p97^R155H/+^ MNs were treated with DMSO, 200 nM or 400 nM Abemaciclib from 8 dpm, and viable cells were determined by live cell staining every 6 days. Abemaciclib of 200 nM improved the viability of p97^R155H/+^ MNs and iso p97^R155H/+^ MNs at 26 dpm (Fig. 4E and Supplementary Fig. S3C). These data suggest that the upregulation of RB1/E2F1 pathway may be related to the cell death of p97^R155H/+^ MNs and CDK4/6 inhibitor can be used to promote MN survival.

To determine whether the dysregulated proteins, we observed were due to the specific genetic background of this particular patient or p97^R155H/+^ mutant, we generated another isogenic pair of MNs derived from an unaffected individual carrying WT p97 and differentiated into MNs using the same method as described previously (Fig. 1D) and harvested at 14 dpm. As shown in Fig. 4F, the upregulation of cell cycle protein (MCM6), cell death associated proteins (p53, γ-H2Ax), E2F1 pathway (E2F1, DHFR), NES, ISL1, were also observed in the isop97 ^R155H/+^ MNs. These results are consistent with p97 ^R155H/+^ and isoWT MNs and indicate that the dysregulation of those proteins was caused by p97 ^R155H/+^ mutant.

### P97 inhibitors relieve p97^R155H/+^-driven neurodegeneration

**Figure 5:**
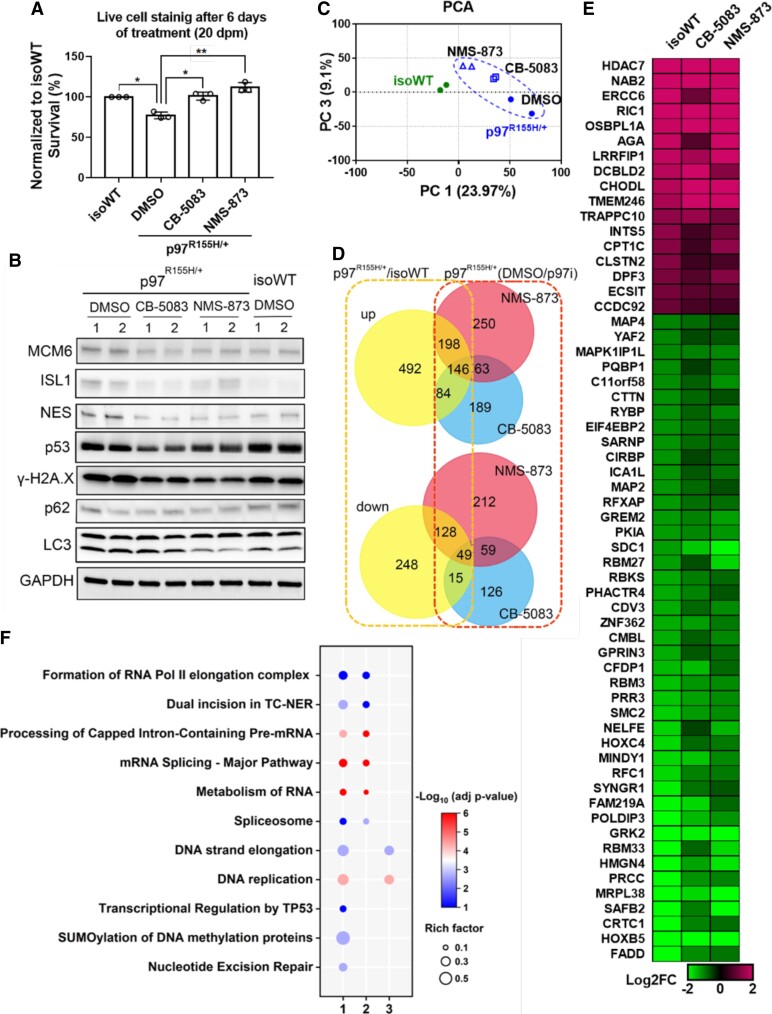
**p97 inhibitors rescue neuron loss and proteomic changes in p97 ^R155H/+^ MNs.** (**A**) Live cell staining and western blots of cells treated with 400 nM of CB-5083 or 400 nM of NMS-873 for 6 days shows reduced neuron loss (A, each data point represents an independent differentiation of the same iPSC line, *n* = 3, *: *P* < 0.05, ** *P* < 0.001 according to unpaired *t*-test.) and reversal of dysregulated protein levels (**B**) in p97^R155H/+^ MNs at 20 dpm. FC indicates the average fold change. (**C**) PCA of proteomics showing the separation between isoWT MNs treated with DMSO and p97^R155H/+^ MNs treated with DMSO or p97 inhibitors. (**D**) Venn diagram showing that p97 inhibitor treatments prevent changes in proteins which were elevated (up) or reduced (down) in D20 p97 MNs. Numbers inside Venn diagram indicate the DEPs in different conditions. (**E**) Heatmap showing that DEPs with |Log2FC|>1 were not seen upon treatment with p97 inhibitors in D20 p97^R155H/+^ MNs. (**F**) Functional enrichment analysis on DEPs identified from D20 p97^R155H/+^ MNs (1), and the DEPs which are reversed (2) or not reversed (3) by p97 inhibitors.

p97 has been implicated in cell cycle regulation and DNA replication,^56,57^ inhibition of p97 downregulates CCND1^58^ and blocks p97 disease mutant phenotypes in *Drosophila.*^26^ We thus tested whether two potent p97 inhibitors rescue p97^R155H/+^ MNs from neurodegeneration. We treated the p97^R155H/+^ and iso p97^R155H/+^ MNs with CB-5083 and NMS-873 at 14 dpm. After 6 days of treatment, both CB-5083 and NMS-873 significantly reduced the loss of MNs (Fig. 5A and Supplementary Fig. S4D). In addition, the treatments reversed increases in ISL1, NES, MCM6, p53, γ-H2Ax in p97^R155H/+^ MNs (Fig. 5B, Supplementary Fig. S5A).

To reveal the global effects of p97 inhibitors on p97^R155H/+^ MNs, proteomic analysis was performed on the D20 MNs after 6 days of treatment with DMSO, CB-5083 or NMS-873. Principal component analysis of proteomic data revealed a clear separation between isoWT and p97^R155H/+^ MNs along principal component 1 (PC1). Treatment with p97 inhibitors reduced the distance between isoWT and p97^R155H/+^ MNs along PC1 (Fig. 5C). We identified 1360 DEPs (*P* < 0.05) in D20 MNs treated with DMSO (Supplementary Table S10). Consistent with our western blot results, proteomics analysis revealed that both CB-5083 and NMS-873 reduced Tau and MCM6 in p97^R155H/+^ MNs (Supplementary Fig. S5B). 428 of the 920 upregulated proteins were reduced and 192 of the 440 downregulated proteins were increased after the treatment with CB-5083 or NMS-873 (Fig. 5D). To further clarify the cellular effects of p97 inhibitors, we listed the most significantly affected DEPs, with | log_2_ (Fold Change) >1 and adjust *P*-values < 0.2, which were corrected to WT level by CB-5083 or NMS-873 (Fig. 5E). Functional enrichment analysis revealed that deregulation of cellular pathways in D20 p97^R155H/+^ MNs are involved in DNA repair and replication, and metabolism of RNA related pathways (Fig. 5F, 1). Surprisingly, cell cycle-related pathways were enriched only at D14 (Fig. 3) but not at D20. Of the 46 dysregulated cell cycle proteins identified from D14 MNs, only 16 exhibited increases in D20 p97^R155H/+^ MNs. The fold change in those 16 proteins in D20 MNs is lower than that in D14 MNs (Supplementary Fig. S5C). The dysregulated proteins which were reversed by p97 inhibitors are linked to RNA metabolism associated pathways (Fig. 5F, 2), and the DEPs which were not corrected by p97 inhibitors are linked to DNA replication (Fig. 5F, 3).

## Discussion

Mutations in p97 cause IBMPFD, a rare multisystem degenerative human disorder that afflicts skeletal muscle, bone and brain.^59,60^ One-third of IBMPFD patients will develop FTD and approximately 10% display features of ALS.^4,9,60^ Currently, there is no cure for IBMPFD patients. Zhang *et al.*^26^ reported that p97 inhibitors can relieve phenotypes, including mitochondrial defects, caused by p97 mutants in adult Drosophila muscle. However, whether p97 inhibitors can prevent neurodegeneration of IBMPFD patient cells was unclear. In addition, the molecular mechanisms underlying neurodegeneration seen in p97 disease mutants is uncertain. Furthermore, published studies using iPSC-derived MNs or cortical neurons from pathogenic p97 mutants, R155C and R191Q, to compare with healthy controls.^22,61–63^ Recently, Harleyet al used the same lines to demonstrate the D2 selective p97 inhibitor, ML240,^64,65^ can rescue mislocalization of TDP-43 and FUS in iPSC-derived MNs from patients with p97^R155C/+^ and p97^R191Q/+^.^66^ To the best of our knowledge, no published studies have used patient cells carrying the most common pathogenic mutation R155H and used isogenic control to validate their results.

p97^R155H/+^ is the most frequently identified p97 disease mutant in IBMPFD patients.^60^ Therefore, we focused on a patient harbouring p97^R155H/+^ and generated isogenic WT controls utilizing CRISPR/Cas9 to exclude the effects of genetic background variation.^67^ In addition, we also made p97^R155H/+^ from a healthy control WT p97 line. No difference was seen in some specific markers of each stage during differentiation, and we observed difference in markers, NES and ISL1, in both RNA and protein levels. We identified a phenotype in mature p97^R155H/+^ MNs. The p97^R155H/+^ MNs displayed a slight reduction in LC3 and increases in p53 as well as γ-H2Ax (Fig. 2E). Our findings suggest the DNA damage response are involved in p97 disease mutant-driven neurodegeneration. Similar to our results, Hall et al. have reported that the p97 mutants R191Q and R155C impair the viability of human-induced MNs.^22^ The cytoplasmic mislocalization of TDP-43 and ubiquitin have been observed in p97 mutant neurons.^12,68^ However, we did not observe cytoplasmic mislocalization of TDP-43, increases in poly-ubiquitinated proteins (k48) or changes in UPR-linked genes in our mature p97^R155H/+^ MNs.

Our data do not show strong dysregulation of wo major protein homeostasis pathways in the iPSC-derived MN model by comparing two isogenic pairs, we instead observed another known role of p97 in regulation of cell cycle is affected by R155H/+ p97 mutation. Future work will reveal the relative contributions of these dysregulations for promotion of MN death. We also observed a FDA-approved CDK4/6 inhibitor, Abemaciclib, can rescue MN death, further supporting the dysregulation of cell cycle in our MN model.

The pathological features of IBMPFD vary among patients and the age of diagnosis indicates that FTD linked to p97 mutation can arise anywhere between 30 and 86 years of age.^9^ These data suggest that the presence of a molecular stress response and pathological markers are culture duration and genetic background dependent in induced MNs.

Cellular functions known to be disrupted in p97 mutant neurons include altered axonal transport of mitochondria,^69^ disrupted synapse formation, decreased ADP/ATP translocation across the mitochondrial membrane and impaired energy metabolism.^22,61^ By performing proteomic and transcriptomic analysis on mature MNs, we systematically explored the dysregulation of proteins and genes in p97^R155H/+^ MNs. Cellular pathways, biological processes and cellular organelles that are potentially impaired as a result of mutation were identified. Interestingly, our data also revealed that p97 mutation leads to cell cycle deregulation in induced MNs. A number of cell cycle genes are upregulated at both the protein and RNA levels. In particular, the RB1/E2F1 pathway is upregulated in p97^R155H/+^ MNs. When nearly 30% MNs were lost following extended maturation culture, the increases in cell cycle proteins abated. Recent research suggests that anomalous cell cycle activation without subsequent induction of mitosis eventually results in apoptosis in neurons.^52^ The dynamic regulation of cell cycle protiens was captured specifically on Days 14 and 20 (Supplementary Fig. S5C). Some of the upregulated protiens were only observed at Day 14 but not at Day 20 and the DNA damage response proteins, p53 and γ-H2Ax, were only downregulated at day 20. One of the possible speculations is that molecular changes linked to delayed cell cycle exit because of this dynamic regulation in p97 mutant MNs. Alterations in the RB1/E2F1 pathway have been observed in ALS neurons *in vivo* and are thought to contribute to neuron cell death in this disorder.^70,71^ Our data provide direct evidence that p97 disease mutants affect the cell cycle in mature MNs and indicates that cell cycle activation may be a cause of p97^R155H/+^-linked MN death.

Increased ATPase activity and altered cofactor binding are seen for the p97^R155H/+^ compared with WT p97. These changes may underlie IBMPFD pathogenesis.^24,25^ ATPase activity of p97 is essential in maintaining CCND1, a critical regulator of the RB1/E2F1 pathway, and inhibition of p97 promotes the degradation of CCND1.^58^ Our data showed that the treatments with either an ATP-competitor or allosteric p97 inhibitor, CB-5083 and NMS-873 respectively, relieves phenotypes in p97^R155H/+^ MNs, including reducing cell death and reducing changes in markers linked to disease pathology (NES, p53 and γ-H2Ax). Proteomic analyses show that nearly half of the dysregulated proteins in p97^R155H/+^ MNs were altered by p97 inhibitors. These include proteins that are linked to RNA metabolism and splicing-related pathways. Our data are consistent with work indicating that ML240 and NMS-873 rescue muscle phenotypes in *Drosophila* harbouring p97 disease mutants.^26^

Taken together, we have made p97^R155H/+^ and isogenic iPSC lines that can be differentiated into disease-relevant cells. Our results suggest that p97 inhibitors and a Cdk4/6 inhibitor are a promising treatment for MN disease caused by p97 mutations. Our proteomic analysis suggests that p97 inhibitor treatments affect RNA processing pathways and cell cycle pathways, raising the possibility that p97 inhibitors might rescue TDP-43-related RNA processing. Future work is needed to validate our findings in different p97 mutants’ patient lines and in animal models. Our established proteomic approaches to identify disease markers and to monitor effect of p97 inhibitors will be excellent tool for these future studies.

## Supplementary Material

fcac176_Supplementary_Data

## Abbreviations

ALS =: familial amyotrophic lateral sclerosis
AP =: alkaline phosphatase
dpi =: Days post induction
dpm =: Days post maturation (dpm)
NEPs =: neuroepithelial progenitors
FC =: fold change
RFLP =: fragment length polymorphism
IBMPFD =: inclusion body myopathy associated with Paget disease of bone and frontotemporal dementia
iPSCs =: induced pluripotent stem cells
isoWT =: isogenic wild type
MN =: motor neuron
MNPs =: motor neuron progenitors
PHF-tau =: paired helical filaments tau
qPCR =: quantitative RT-PCR
UPR =: unfolded protein response
