# Supplementary material for: P97/VCP ATPase inhibitors can rescue p97 mutation-linked motor neuron degeneration
Source: Brain Commun. 2022 Jul 6;4(4):fcac176. doi: 10.1093/braincomms/fcac176 (PMC9294923; doi:10.1093/braincomms/fcac176)
Supplement: fcac176_Supplementary_Data [file fcac176_supplementary_data.zip › Supplementary_material.pdf]

Supplementary Information

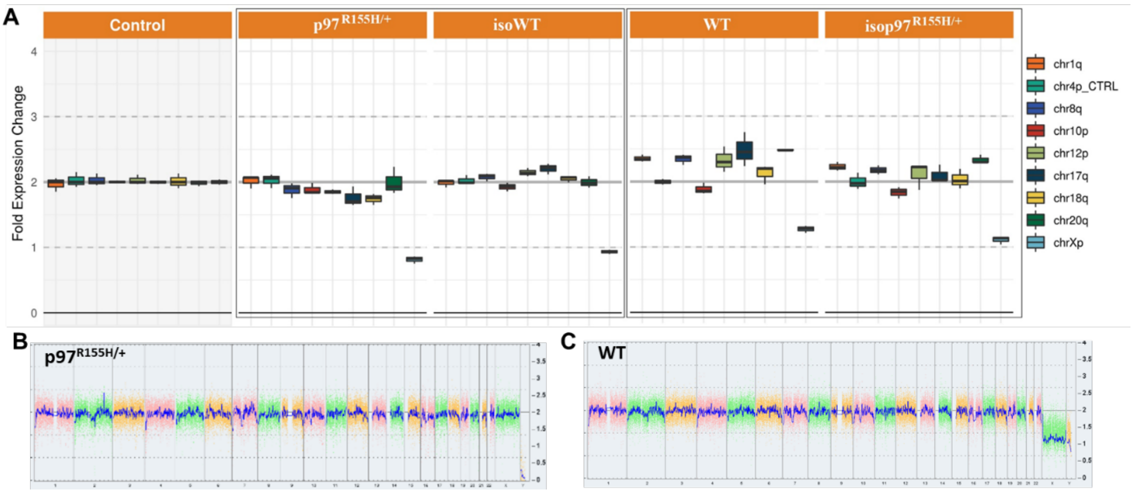

Supplementary Figure 1. Identification of genetic abnormalities in iPSCs. A) The majority of recurrent karyotypic abnormalities reported in iPSCs were detected using quantitative (qPCR) and no gene depletion or amplification was observed in the clones used for differentiation. B-C) Karyotyping results in the p97$^{R155H/+}$ clone: GM21851 (B) and WT clone: GM22246 (C) used for differentiation displayed no karyotypic abnormalities.

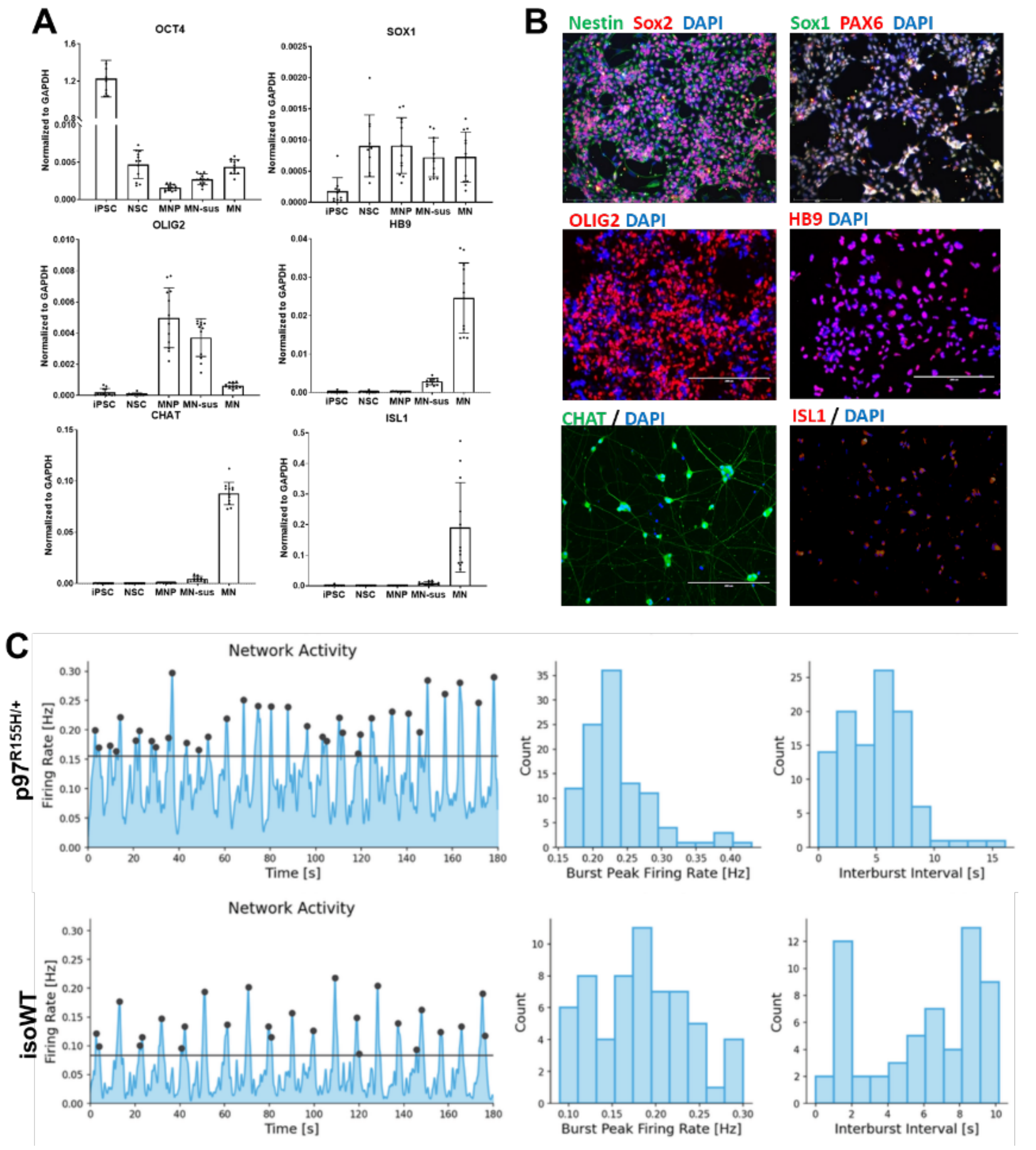

Supplementary Figure 2. iPSCs were induced to form functional MNs. A) qPCR results displaying changes in defined biomarkers during the differentiation. Each data point represents an independent differentiation of both mutant and wild-type iPSC lines. B) IF shows expression of Nestin, Sox1, Sox2 and PAX6 in NEPs, Olig2 in MNPs, HB9 in immature MNs and CHAT, ISL1 in mature MNs. Scale bar is 200 μm. C) Neuron activity recorded by Maxwell displaying the MNs formed network at 14 dpm.

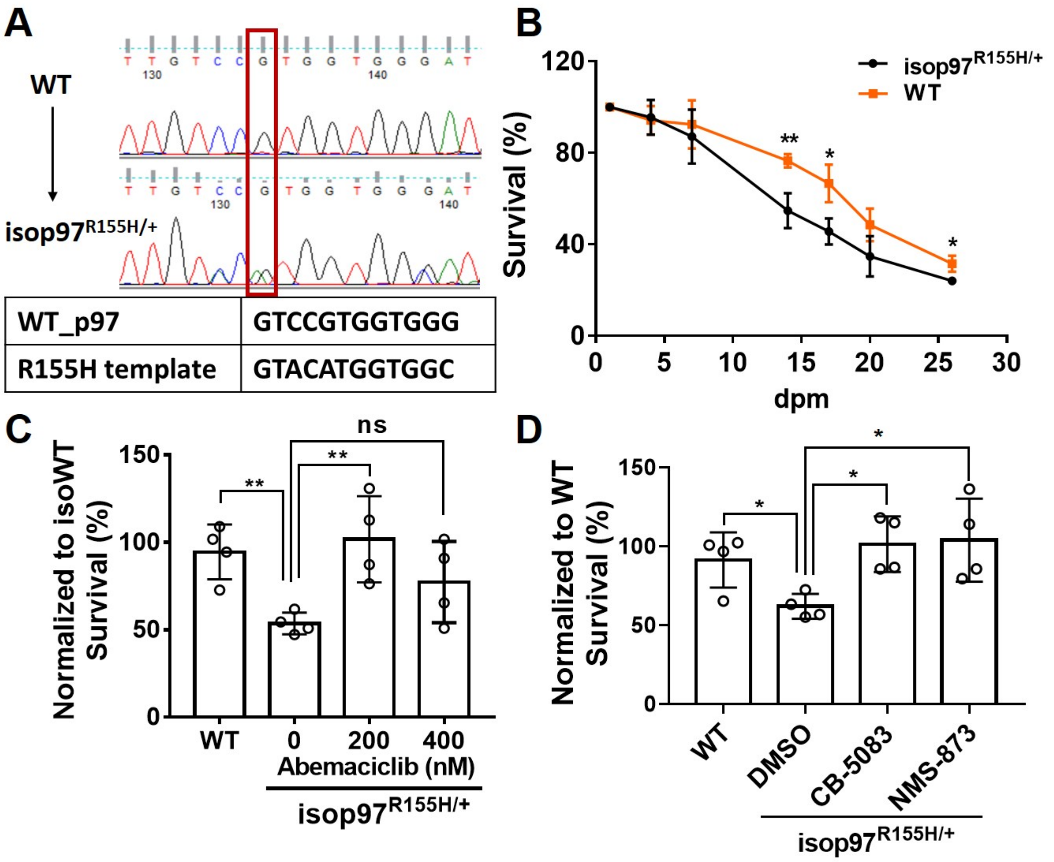

Supplementary Figure 3. Isogenic p97$^{R155H/+}$ iPSC and NN generated from a healthy control line. A) DNA sequence of the isogenic p97$^{R155H/+}$ iPSC. B) Cell survival curves during MN maturation. Data represent mean ± SD, n=3. *: $p < 0.05$, **: $p < 0.01$ according to unpaired t-test. C) Cell survival results of MNs were treated with DMSO, 200 nM or 400 nM Abemaciclib. Live cell staining was performed at 26 dpm, total cell numbers were counted and normalized to isoWT MNs, n=4, **: $p < 0.01$ according to unpaired t-test. D) Live cell staining of cells treated with 100 nM of CB-5083 or 100 nM of NMS-873 for 6 days shows reduced neuron loss, N=4, *: $p < 0.05$ according to unpaired t-test.

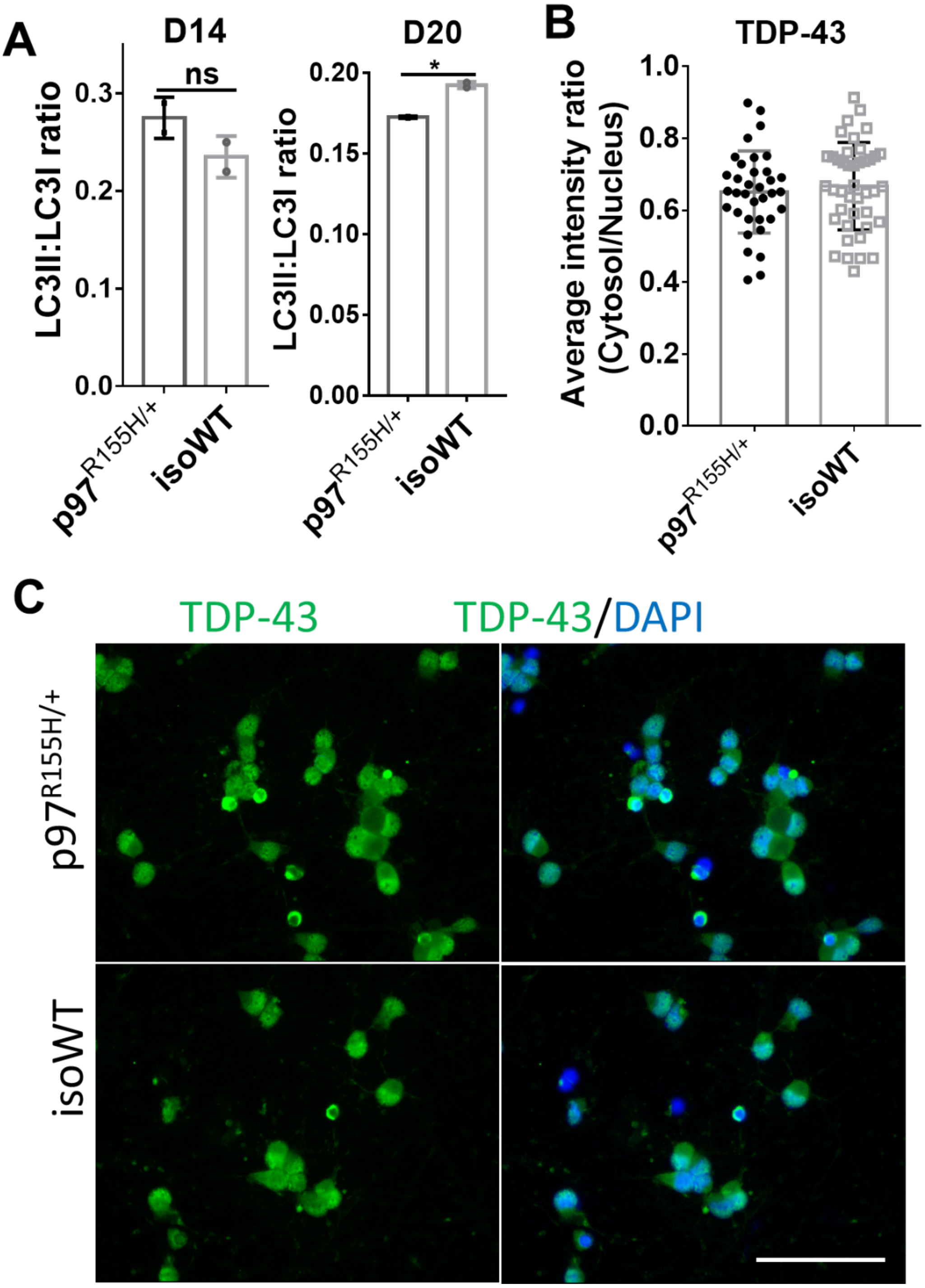

Supplementary Figure 4. LC3II:LC3I ratio (A) and IF staining displaying that the TDP-43 exhibited no difference between p97$^{R155H/+}$ and isoWT MNs (B-C). Scale bar indicates 50μm.

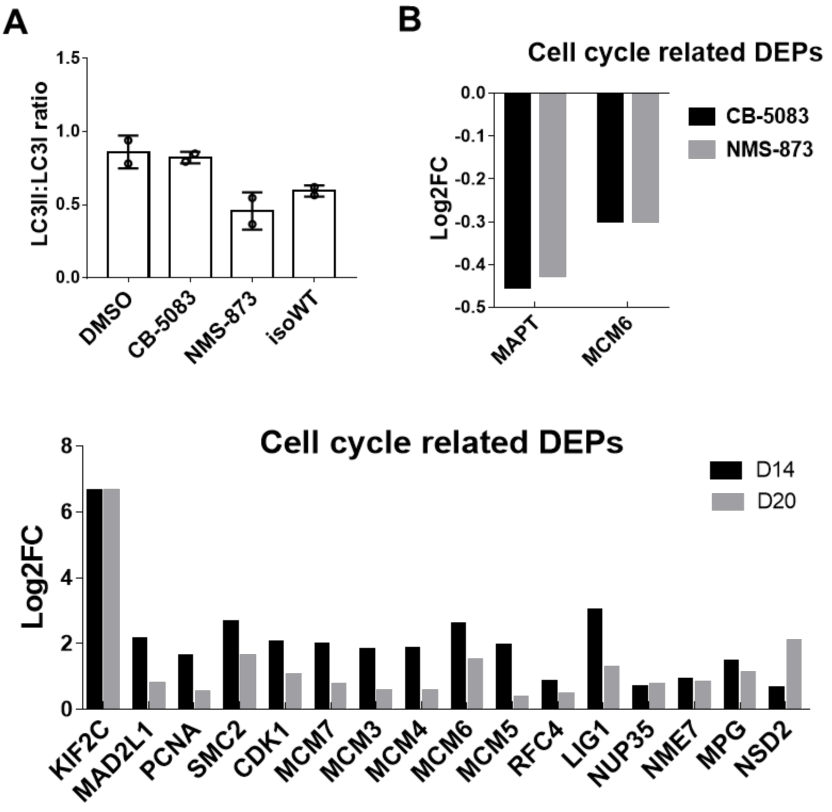

Supplementary Figure 5. Protein changes detected by LC/MS/MS. A) LC3II:LC3I ratio of MNs after the treatments with DMSO or p97 inhibitors. B) MAPT (Tau) and MCM6 were reduced after 6 days of treatment with p97 inhibitors. C) The abundance of cell cycle proteins decreased following the extended maturation culture.

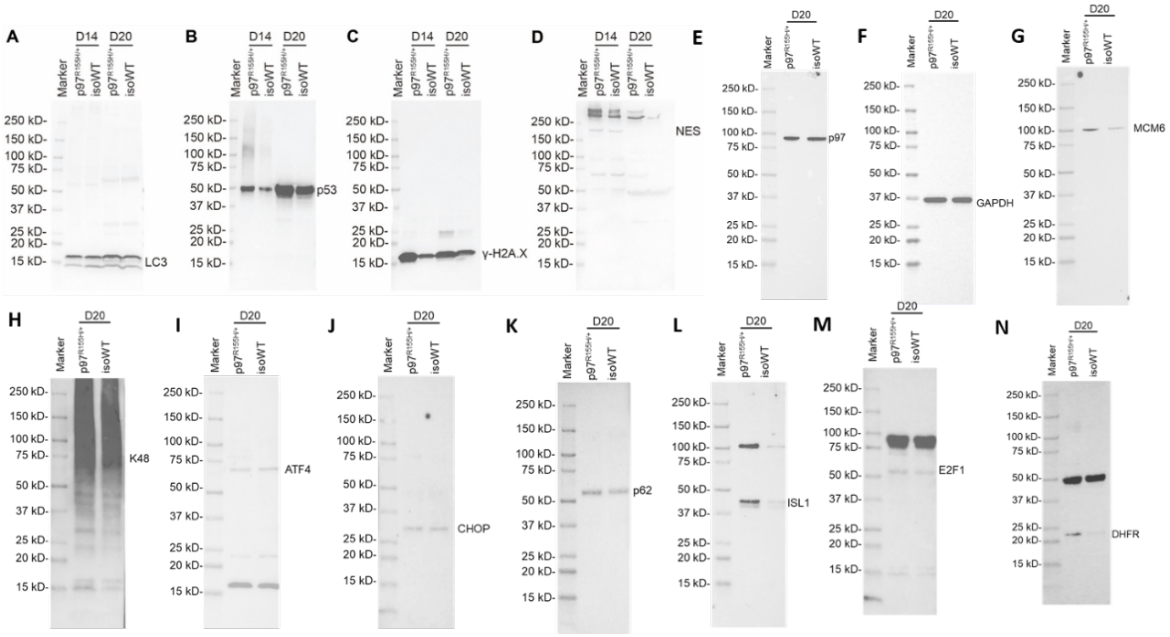

Supplementary Figure 6. Uncropped western blot images.

Supplemental Tables

Table S1. Oligonucleotide sequences for gene editing and PCR

Name	Sequence
Guide RNA	5'- CCACAGCACGCATCCCACCA -3'
H155R-Reverse Complement	5'- ATCTGTTTCCACCACTTTGAACTCCACAGCACGCATGCCACCA CGTACAAGAAAAATGTCTCCTGCGAGAGCAAACAGTA -3'
R155H-Reverse Complement	5'- ATCTGTTTCCACCACTTTGAACTCCACAGCACGCATGCCACCA TGTACAAGAAAAATGTCTCCTGCGAGAGCAAACAGTA -3'
VCP-Ex5-F	5'- TGGAGTTGGGGAGAGGTAGGG -3'
VCP-Ex5-R	5' AAAATCGGATACTGGAATCAGGGAGA -3'

Table S2 qPCR probes used in this study

Target	Catalog number
GAPDH	Hs02786624_g1
OCT4	Hs00999634_gH
PAX6	Hs01088106_g1
NES	Hs04187831_g1
SOX1	Hs01057642_s1
OLIG2	Hs00300164_s1
HB9	Hs00232128_m1
ISL1	Hs00158126_m1
CHAT	Hs00758143_m1

Table S3 Primary antibodies used in this study

Antibodies	Catalog number	RRID	Dilution
CHAT	PA5-29653	AB_2547128	1:200 (IF)
ISL1	PA5-27789	AB_2545265	1:5000 (WB); 1:200 (IF)
OLIG2	sc-293163	Ref: [1, 2]	1:250 (IF)
HB9	DSHB 81.5C10	AB_2145209	1:40 (IF)
TUJ1	BioLegend 801202	AB_10063408	1:500 (IF)
TDP-43	10782-2AP	AB_615042	1:100 (IF)
k48	Boston Biochem A-101	AB_10699867	1:1000 (WB)
ATF4	SC-200	AB_2630429	1:400 (WB)
CHOP	CST2895	AB_2089254	1:250 (WB)
p62	M162-3	AB_1279299	1:3000 (WB)
LC3	PM036	AB_2274121	1:3000 (WB)
p-TAU	ab92676	AB_10561457	1:1500 (WB); 1:100 (IF)
TAU	ab80579	AB_1603723	1:1000 (WB)
p53	sc-126	AB_628082	1:200 (WB)
γ-H2AX	ab26350	AB_470861	1:3000 (WB)
p97	MA3-004	AB_2214638	1:3000 (WB)
GAPDH	CST2118	AB_561053	1:5000 (WB)
Filamin 1	sc-17749	AB_627606	1:1000 (WB)
MCM6	sc-393618	AB_2885187	1:500 (WB)
NES	MA1-110	AB_2536821	1:1000 (WB)
HSP 47	sc-5293	AB_627757	1:200 (WB)
pRB1	sc-377528	Ref: [3, 4]	1:200 (WB)
E2F1	sc-251	AB_627476	1:200 (WB)
CCND1	sc-8396	AB_627344	1:100 (WB)
DHFR	PA5-30992	AB_2548466	1:1000 (WB)
